# Nonlinear Carrier Dynamics and Optical Response of Lanthanum-Doped Barium Stannate

**DOI:** 10.3390/nano16181156

**Published:** 2026-09-15

**Authors:** Viktoriia E. Babicheva, Heungsoo Kim, Evgeniya H. Lock

**Affiliations:** 1 Department of Electrical and Computer Engineering, University of New Mexico, MSC01 1100, 1 University of New Mexico, Albuquerque, NM 87131, USA; 2 Naval Research Laboratory, 4555 Overlook Ave, Washington, DC 20375, USA

**Keywords:** lanthanum-doped barium stannate, epsilon-near-zero materials, transparent conducting oxides, nonlinear optics, plasma frequency tuning, ultrafast modulation

## Abstract

Lanthanum-doped barium stannate (LBSO) is an emerging transparent conducting oxide with high carrier mobility and tunable plasmonic properties in the infrared regime. In this work, we investigate the nonlinear electromagnetic response of LBSO using a linearized collisionless Boltzmann model that captures the field-induced modification of the carrier distribution function. We experimentally realize LBSO thin films with controlled carrier concentration and optical properties tailored for epsilon-near-zero operation. Analytical expressions for the nonlinear plasma frequency and permittivity reveal their dependence on carrier concentration, temperature, and optical excitation. An excitation-driven reduction in the effective plasma frequency is accompanied by significant changes in the permittivity spectrum. The results demonstrate that the LBSO metasurface exhibits strong optical tunability under near-infrared excitation, with significant modulation of its reflection and transmission characteristics. These findings provide physical insight into the nonlinear carrier dynamics in LBSO and establish a framework for designing tunable photonic devices based on transparent conducting oxides.

## 1. Introduction

The advancement of photonic modulation technologies has played a central role in the evolution of modern optical systems, enabling progress in communications, sensing, and information processing applications [1,2,3]. In recent years, significant attention has been paid to incorporating nonlinear optical phenomena into photonic platforms to achieve enhanced functionality beyond that of conventional modulators. Transparent conducting oxides (TCOs) have emerged as promising materials for this purpose due to their distinctive electrical and optical characteristics [4,5,6,7,8,9,10]. Materials, such as indium tin oxide (ITO) and aluminum-doped zinc oxide (AZO), combine high electrical conductivity with broadband optical transparency, allowing strong light–matter interactions and pronounced optical nonlinearities while maintaining relatively low optical losses [11,12,13]. Most importantly, the nonparabolicity of the conduction band in TCOs provides a strong mechanism for the intrinsic nonlinear electromagnetic response through field-dependent carrier effective mass and velocity [14,15,16,17,18,19,20,21,22]. TCOs exhibit epsilon-near-zero (ENZ) behavior when their permittivity crosses zero near the plasma frequency, leading to strongly enhanced and spatially delocalized electromagnetic fields, pronounced nonlinear optical response due to intensified local field amplitudes, and a transition to a regime where phase variation across subwavelength distances becomes strongly suppressed [23,24,25,26,27,28].

Among emerging TCOs, lanthanum-doped barium stannate (La:BaSnO_3_, LBSO) has attracted considerable interest due to its exceptional electronic, optical, and thermal properties [18,29,30,31,32,33,34,35]. LBSO is a cubic perovskite oxide that exhibits high carrier mobility, excellent optical transparency, and substantial electrical conductivity, making it a promising material for transparent electronics, optoelectronic devices, and infrared photonic applications [36]. Pulsed laser deposition (PLD) has emerged as a particularly effective technique for the growth of high-quality thin films due to its precise control over stoichiometry, crystallinity, and defect concentration [29,30,37,38,39,40,41,42,43,44,45,46,47,48,49]. As a result, PLD-grown LBSO films routinely exhibit excellent structural quality and enhanced electronic and optical performance, making them well-suited for investigating fundamental transport and photonic phenomena. Furthermore, LBSO behaves as a refractory ENZ material and has demonstrated remarkable thermal stability in air at temperatures exceeding 1000 °C, as well as strong resistance to intense ultraviolet laser irradiation [50,51]. These characteristics distinguish LBSO from conventional TCOs and expand its applicability in harsh-environment photonic systems.

Nonlinear optical phenomena in TCOs offer a pathway to dynamic manipulation of electromagnetic waves via mechanisms such as intensity-dependent refractive-index modulation, harmonic generation, and parametric frequency conversion [52,53]. TCOs exhibit an ultrafast optical response governed by rapid free-carrier dynamics in a Drude-like plasma, where femtosecond-to-picosecond changes in carrier energy distribution and scattering rates lead to swift modulation of the complex permittivity, producing transient refractive-index variations and strongly time-dependent nonlinear optical behavior [23,54,55]. The nonlinear response of the TCO can be described within a collisionless Boltzmann framework for conduction electrons, in which field-induced modifications of the carrier distribution alter the effective plasma frequency and dielectric permittivity. These effects enable ultrafast optical modulation and provide new opportunities for tunable infrared and terahertz photonic devices. Although the nonlinear optical response of LBSO remains relatively unexplored, its combination of high carrier density and low-loss optical behavior suggests considerable potential for integrated nonlinear photonics [23,56,57,58,59,60]. This nonparabolicity-driven carrier-dynamics mechanism has not yet been explored in LBSO, despite its expected relevance in the highly doped regime.

In this paper, we investigate the nonlinear LBSO response and demonstrate the possibility of effective optical control of this material. We fabricate and characterize LBSO thin films with controlled material properties, enabling systematic investigation of their ENZ response. The analysis is based on a linearized collisionless Boltzmann description of conduction electrons, which enables evaluation of field-induced modifications to the carrier distribution and their impact on the material’s optical properties. Analytical expressions are derived for the effective plasma frequency and dielectric permittivity, and their dependence on carrier concentration, temperature, and optical excitation is examined. Our results show that optical-pump-induced plasma-frequency modulation enables efficient switching of the LBSO metasurface response, leading to substantial spectral changes in reflection and transmission. The results obtained provide insight into the mechanisms governing nonlinear carrier dynamics in LBSO and assess its potential for tunable photonic and optoelectronic applications.

## 2. Linearized Collisionless Boltzmann Model

The optical dielectric response of LBSO can be approximated using a combined Drude–Lorentz and Tauc–Lorentz model. In the infrared range, the permittivity is

(1) $$\varepsilon \left(\omega \right)=\varepsilon_{\infty }-\frac{\omega_{p}^{2}}{\omega^{2}+i\gamma \omega },$$

where $\varepsilon_{\infty }$, $\omega_{p}$, and γ are the high-frequency background permittivity, plasma frequency, and damping rate, respectively. Within this framework, the effective electron mass is $m^{*}=ne^{2}/\omega_{p}^{2}$, with the carrier density *n* coinciding with the one obtained in electrical Hall measurements, and $m^{*}$ increasing from $0.19\;m_{0}$ to $0.36\;m_{0}$ ($m_{0}$ is the mass of the free electron) as *n* increases from $0.8\times 10^{19}$ to $7.6\times 10^{20}\;{\mathrm{cm}}^{-3}$ [18]. In what follows, $\omega_{p}$ is dependent on the temperature of the electrons *T* while *n* remains fixed, and thus $m^{*}\left(T\right)=ne^{2}/\omega_{p}{\left(T\right)}^{2}$, while *m* is introduced as an effective mass of electron at the conduction band minimum.

**Plasma Frequency in Nonparabolic Bands.** Plasma-frequency tuning can arise from changes in the carrier concentration and, more generally, from changes in the effective carrier mass. In the present analysis, the excitation-induced change in $\omega_{p}$ is primarily associated with the modification of the carrier distribution and its corresponding effective response. In LBSO under intraband excitation, *n* is conserved; thus, the observed modulation of $\omega_{p}$ originates from effects of the band structure. This behavior is attributed to conduction-band nonparabolicity, where the effective mass increases with carrier energy [14,15,16]. The dispersion of electrons with energy *E* is modeled as

(2) $$\frac{\hbar^{2}k^{2}}{2m}=E+\frac{E^{2}}{E_{g}},$$

where $1/E_{g}$ quantifies the nonparabolicity of the conduction band through empirical fitting and *k* denotes the general wavevector (with the Fermi wavevector $k_{F}={\left(3\pi^{2}n\right)}^{1/3}$).

The derivation follows from the collisionless Boltzmann equation

(3) $$\frac{\partial \delta f}{\partial t}+v\cdot \frac{\partial \delta f}{\partial r}+F\cdot \frac{\partial f_{0}}{\hbar \partial k}=0,$$

together with the self-consistent electrostatic relations $E=-\nabla \phi ,\;\nabla^{2}\phi =e\;\delta n/\varepsilon .$ Here, f(r,k,t) denotes the electron distribution function, which is decomposed as $f=f_{0}+\delta f$, where $f_{0}$ is the equilibrium Fermi–Dirac distribution and δf represents the small perturbation induced by the collective electron oscillation.

In Fourier space, this yields

(4) $$\delta f\left(q\right)=i\frac{\partial f_{0}}{\partial E}\frac{e\;E(q)\cdot v}{\omega_{p}-q\cdot v}.$$

The long-wavelength expansion of the dispersion relation gives

(5) $$\omega_{p}^{2}=\frac{e^{2}}{3\pi^{2}}\int_{0}^{\infty }dE\;\frac{m}{\hbar^{2}}\left(1+\frac{2E}{E_{g}}\right){\left[\frac{2m}{\hbar^{2}}\left(E+\frac{E^{2}}{E_{g}}\right)\right]}^{1/2}v^{2}\left(-\frac{\partial f_{0}}{\partial E}\right),$$

with the velocity defined as

(6) $$v=\frac{1}{\hbar }\frac{\partial E}{\partial k}=\frac{\hbar k}{m\left(1+2E/E_{g}\right)}.$$

From the linearized collisionless Boltzmann equation and with the assumption of small-*q* collective oscillations, the plasma frequency in an isotropic nonparabolic band is

(7) $$\omega_{p}^{2}\left(\mu ,T\right)=\frac{e^{2}}{3m\pi^{2}}\int_{0}^{\infty }dE{\left[\frac{2m}{\hbar^{2}}\left(E+\frac{E^{2}}{E_{g}}\right)\right]}^{3/2}{\left(1+\frac{2E}{E_{g}}\right)}^{-1}\left(-\frac{\partial f_{0}\left(\mu ,T\right)}{\partial E}\right).$$

Substituting the dispersion relation leads back to the compact expression for $\omega_{p}^{2}\left(\mu ,T\right)$. In the parabolic limit $E_{g}\to \infty$,

(8) $$\omega_{p}^{2}=\frac{ne^{2}}{m},\;\int_{0}^{\infty }dE{\left(\frac{2m}{\hbar^{2}}E\right)}^{3/2}\left(-\frac{\partial f_{0}}{\partial E}\right)=3\pi^{2}n.$$

**Carrier and Energy Densities.** Ultraviolet pumping can induce significant interband absorption, generating additional free carriers and thereby modifying the carrier density *n* beyond the purely intraband nonlinear regime [11]. However, our model assumes infrared, low-photon-energy excitation, under which interband transitions are negligible and the carrier concentration remains effectively conserved. At finite temperature, carrier conservation determines the chemical potential through

(9) $$n\left(\mu ,T\right)=\frac{1}{\pi^{2}}\int_{0}^{\infty }dE\;\frac{m}{\hbar^{2}}\left(1+\frac{2E}{E_{g}}\right){\left[\frac{2m}{\hbar^{2}}\left(E+\frac{E^{2}}{E_{g}}\right)\right]}^{1/2}f_{0}\left(\mu ,T\right),$$

and the electron energy density is

(10) $$U\left(\mu ,T\right)=\frac{1}{\pi^{2}}\int_{0}^{\infty }dE\;\frac{m}{\hbar^{2}}E\left(1+\frac{2E}{E_{g}}\right){\left[\frac{2m}{\hbar^{2}}\left(E+\frac{E^{2}}{E_{g}}\right)\right]}^{1/2}f_{0}\left(\mu ,T\right).$$

The temperature-dependent plasma dynamics follows from the coupled set μ(T), $\omega_{p}\left(T\right)$, and U(T). Optical pumping increases the electron temperature above 300 K while conserving the carrier density. The excitation-induced energy is ΔU(T)=U(T)−U(300K), defined as the calculated excitation energy density. This mapping enables direct extraction of *T* and $\omega_{p}$ under optical excitation. At high fluence, LBSO reaches *T*∼$10^{4}$ K, producing a substantial reduction in $\omega_{p}$. The reduced carrier density in LBSO yields a heat capacity lower than that of noble metals, enabling higher electron temperatures and faster relaxation dominated by electron–phonon coupling.

## 3. LBSO Film Growth and Characterization

We employ PLD growth of La:BaSnO_3_ grown on MgO substrates. This technique has been shown to produce LBSO of high quality with a fast deposition rate (typically at least 10 times faster than that of molecular beam epitaxy). A KrF excimer laser (Lambda Physik, LPX 305, 248 nm, pulse duration 30 ns, Göttingen, Germany) is focused by a 50 cm focal length lens onto a rotating target at a 45° angle of incidence with a pulse rate of 5 Hz. The energy density of the laser beam is maintained at ∼1.5 J/cm^2^ on the target surface, and the target-to-substrate distance is 5 cm. The LBSO target (2.54 cm in diameter) is acquired from American Elements. First, before deposition, the PLD chamber is pumped down to <$1\times 10^{-2}$ mTorr. For growth, O_2_ is purged through the chamber to keep the required pressure in the range of 20–100 mTorr. In the process, the laser spot is translated across the rotating target throughout deposition to achieve homogeneous ablation of the target material. The sample is likewise rotated at 20 rpm to enhance film thickness uniformity. The sample temperature during deposition is maintained at 750 °C. Subsequently, the chips are cooled down to room temperature at the same O_2_ pressure. The crystalline structures of the LBSO and MgO substrate are analyzed by X-ray diffraction characterization with Cu K$\alpha_{1}$ (Bruker D8). The complex refractive index of the LBSO layer is extracted from spectroscopic ellipsometry measurements at wavelengths from 400 nm to 5 μm (VASE and IR-WVASE, J.A. Woollam).

The properties of LBSO can be readily tailored by variations in the fabrication process, including dopant concentration and deposition conditions. LBSO thin films are grown on mismatched lattice substrates using controlled deposition conditions, allowing systematic tuning of structural strain via partial oxygen pressure during growth (Table 1). Comprehensive material characterization reveals that strain relaxation correlates with enhanced carrier transport, in which reduced in-plane and out-of-plane strain components suppress dislocation-mediated scattering, thereby improving carrier mobility. The systematic increase in $\lambda_{\mathrm{ENZ}}$ with decreasing carrier concentration is consistent with the Drude description, in which the plasma frequency decreases as the free-carrier density is reduced. The small deviations from strict monotonicity observed for samples #5 and #6 are attributed to variations in the film growth conditions and the associated defect and scattering populations. In particular, while the carrier concentration primarily determines the plasma frequency and hence the ENZ wavelength, the damping rate is additionally sensitive to carrier scattering by defects, impurities, structural disorder, and other microstructural features. Consequently, the damping rate need not vary monotonically with carrier concentration.

The solubility of La in BaSnO_3_ depends on the synthesis and growth conditions [61]. For bulk La-doped BaSnO_3_, a solubility limit of approximately 3 at.% has been reported, above which secondary phases such as La_2_Sn_2_O_7_ can form [62,63]. In contrast, higher nominal La concentrations have been incorporated into thin films under nonequilibrium growth conditions [64,65]. The nominal La concentration also does not necessarily correspond to the free carrier concentration, since carrier trapping, compensation, and structural defects can reduce the electrically active fraction of the dopants. Previous transport studies have reported activation ratios below unity, with values of approximately 70% reported for highly crystalline La-doped BaSnO_3_ films [66]. In the present work, the carrier concentration used in the optical analysis is determined experimentally rather than being inferred directly from the nominal La concentration.

**Figure 1:**
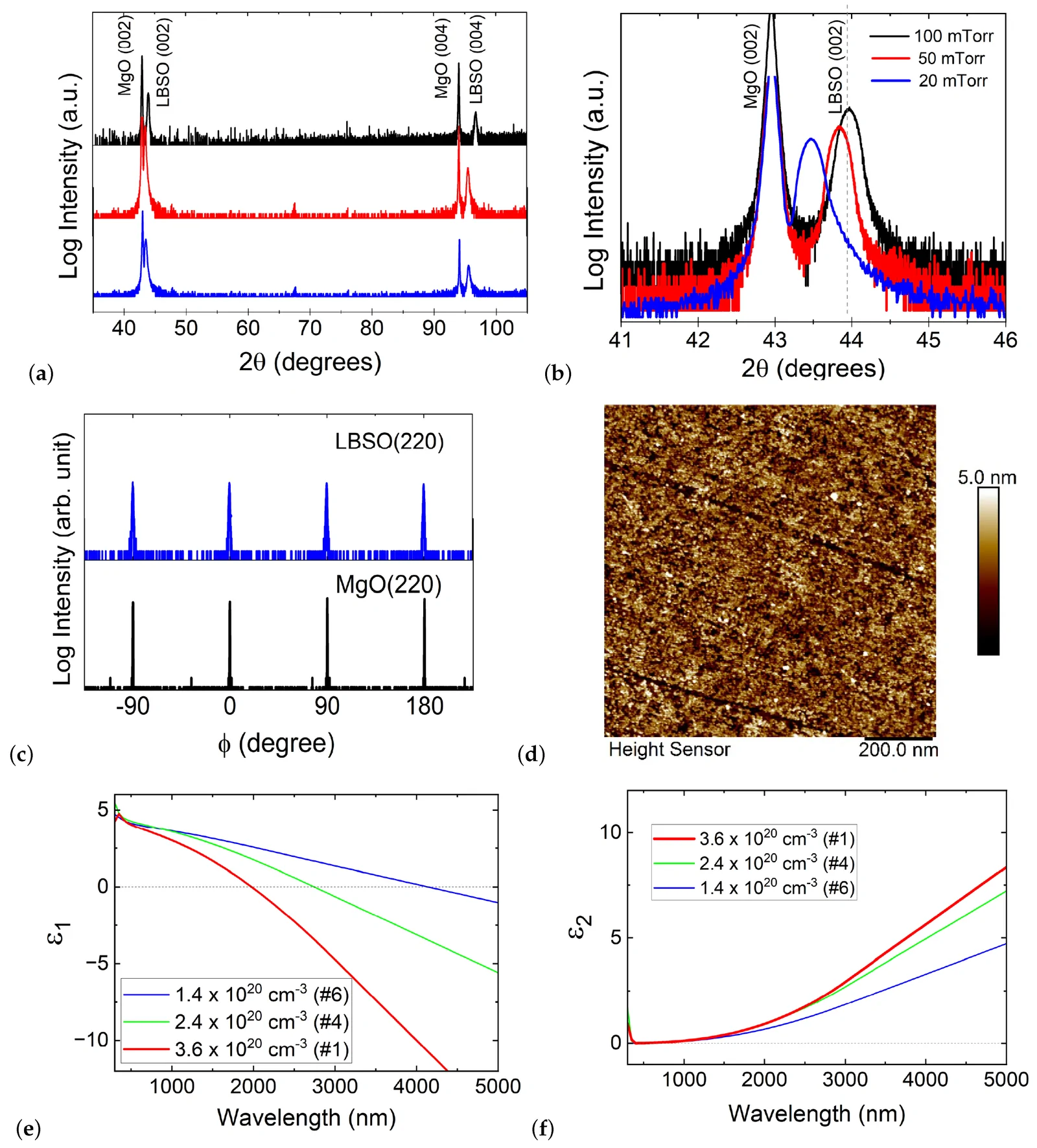
(**a**) X-ray diffraction 2θ scans of LBSO layers grown on (001) MgO with variable O_2_ pressures (100, 50, and 20 mTorr) for samples #1, #4, and #6, respectively, with a) wide 2θ angles (35–105°) and (**b**) zoomed-in analysis 2θ angles (41–46°) near (002) diffraction peaks of MgO and LBSO. The dashed lines show the bulk (002) LBSO peak in panel (**b**). (**c**) X-ray diffraction ϕ scans of (220) MgO and (220) LBSO peaks of the layers grown at 100 mTorr. (**d**) AFM micrograph of LBSO film on MgO grown at 100 mTorr O_2_ pressure (sample #1). (**e**) Real part $\varepsilon_{1}=ℜ\left[\varepsilon \right]$ and (**f**) imaginary part $\varepsilon_{2}=ℑ\left[\varepsilon \right]$ of LBSO permittivity obtained with ellipsometry measurements.

Figure 1a shows the X-ray diffraction 2θ scans of LBSO films grown on MgO substrates at three different O_2_ pressures (20, 50, and 100 mTorr) for samples #6, #4, and #1, respectively. We observe only (00*l*) diffraction peaks from the MgO substrate or LBSO film, indicating highly oriented film growth along the c-axis with no other impurity peaks. The (00*l*) film peaks shift to higher 2θ angles with increasing the O_2_ growth pressure from 20 to 100 mTorr (see Figure 1b). With a cube-on-cube relationship, the X-ray diffraction ϕ scan verifies that the LBSO layers are epitaxially formed on the MgO substrate (Figure 1c). The surface morphology of the LBSO film is measured using an atomic force microscope (Bruker Dimension Icon, Santa Babara, USA, Figure 1d). The film shows a uniform grain distribution with a grain size range of 20–40 nm and smooth surface morphology (rms roughness of 0.6 nm).

Optical characterization using spectroscopic ellipsometry combined with Lorentz–Drude modeling reveals a clear dependence of the complex permittivity response and the spectral position of the ENZ point on growth conditions. Figure 1e,f shows the optical permittivity values (ℜ[ε] and ℑ[ε]) for LBSO films with various carrier densities derived from spectroscopic ellipsometry, where ℜ[ε] corresponds to the real part of the LBSO permittivity, and ℑ[ε] corresponds to the imaginary part of the LBSO permittivity. The $\varepsilon_{1}$ decreases, and the $\varepsilon_{2}$ increases with wavelength, which is in good agreement with the Drude model. The $\lambda_{\mathrm{ENZ}}$ is found to shift towards longer wavelengths from the 2000 nm to 4000 nm range as the O_2_ deposition pressure decreases from 100 mTorr to 20 mTorr. The combined structural, electrical, and optical analyses establish a direct link between film growth-induced strain engineering and the tunable plasmonic response of LBSO thin films.

**Figure 2:**
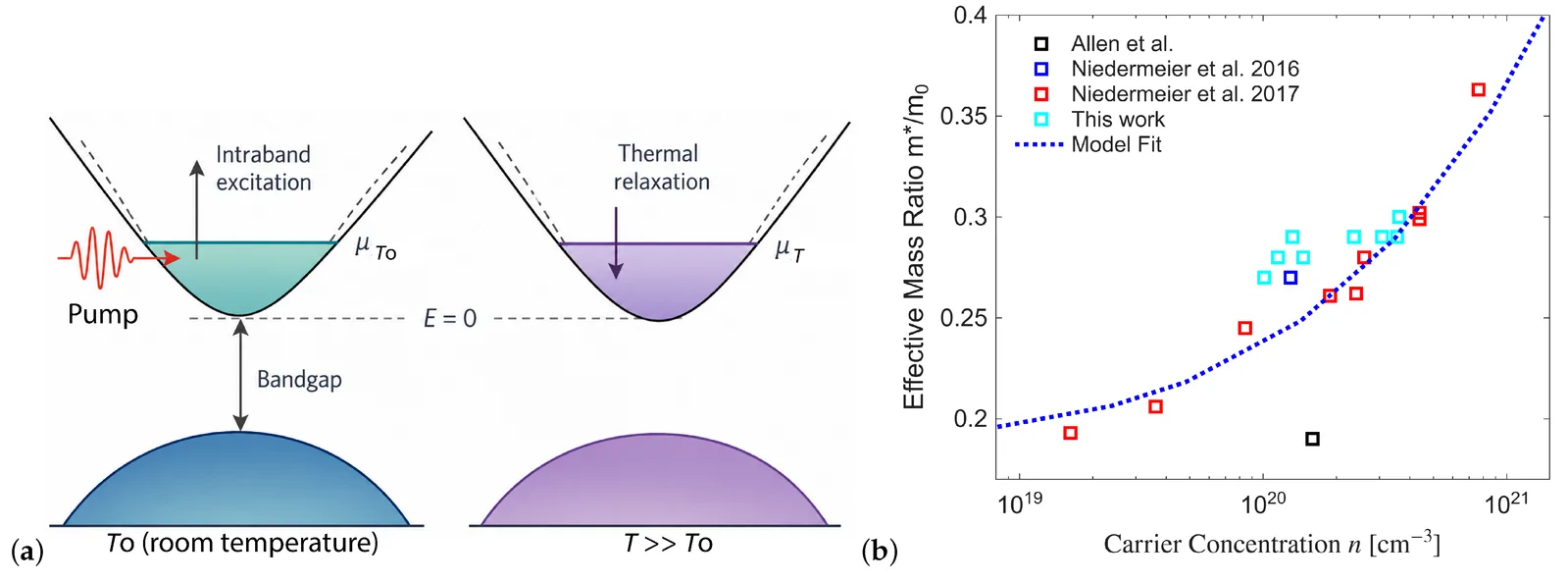
(**a**) Schematic of the electron populations and carrier transitions associated with near-infrared intraband excitation. The conduction band has nonparabolic dispersion (solid lines), and the parabolic case is shown for comparison (dashed lines). (**b**) Increase in LBSO electron effective mass as a function of carrier concentration *n*. In our analysis, the data is extracted via the Drude model (Table 1). For comparison, effective mass values are shown for what has been reported previously: Allen et al. [32], Niedermeier et al., 2016 [36], and Niedermeier et al., 2017 [18]. The fit model uses values $1/E_{g}=0.37\;{\mathrm{eV}}^{-1}$ and $m=0.19\;m_{0}$.

The typical parameters for TCOs are the following: For ITO, these are $m=0.26\;m_{0}$ and $1/E_{g}=0.42\;{\mathrm{eV}}^{-1}$ [16] (with earlier reports of $m=0.30\;m_{0}$ and $1/E_{g}=0.18\;{\mathrm{eV}}^{-1}$ [17]). For AZO, these are $m=0.24\;m_{0}$ and $1/E_{g}=0.27\;{\mathrm{eV}}^{-1}$ [15], and AZO is substantially less nonparabolic than ITO. For Ga:ZnO, these parameters are $m=0.28\;m_{0}$ and $1/E_{g}=0.14\;{\mathrm{eV}}^{-1}$ [17,67,68,69]. For LBSO, recently reported values of the effective mass and degree of nonparabolicity of the conduction band are $m=0.19\;m_{0}$ and $1/E_{g}=0.37\;{\mathrm{eV}}^{-1}\;$[18]. Our results agree with the reported values (Figure 2), and we use them further in our calculations.

## 4. Nonlinearity

**Figure 3:**
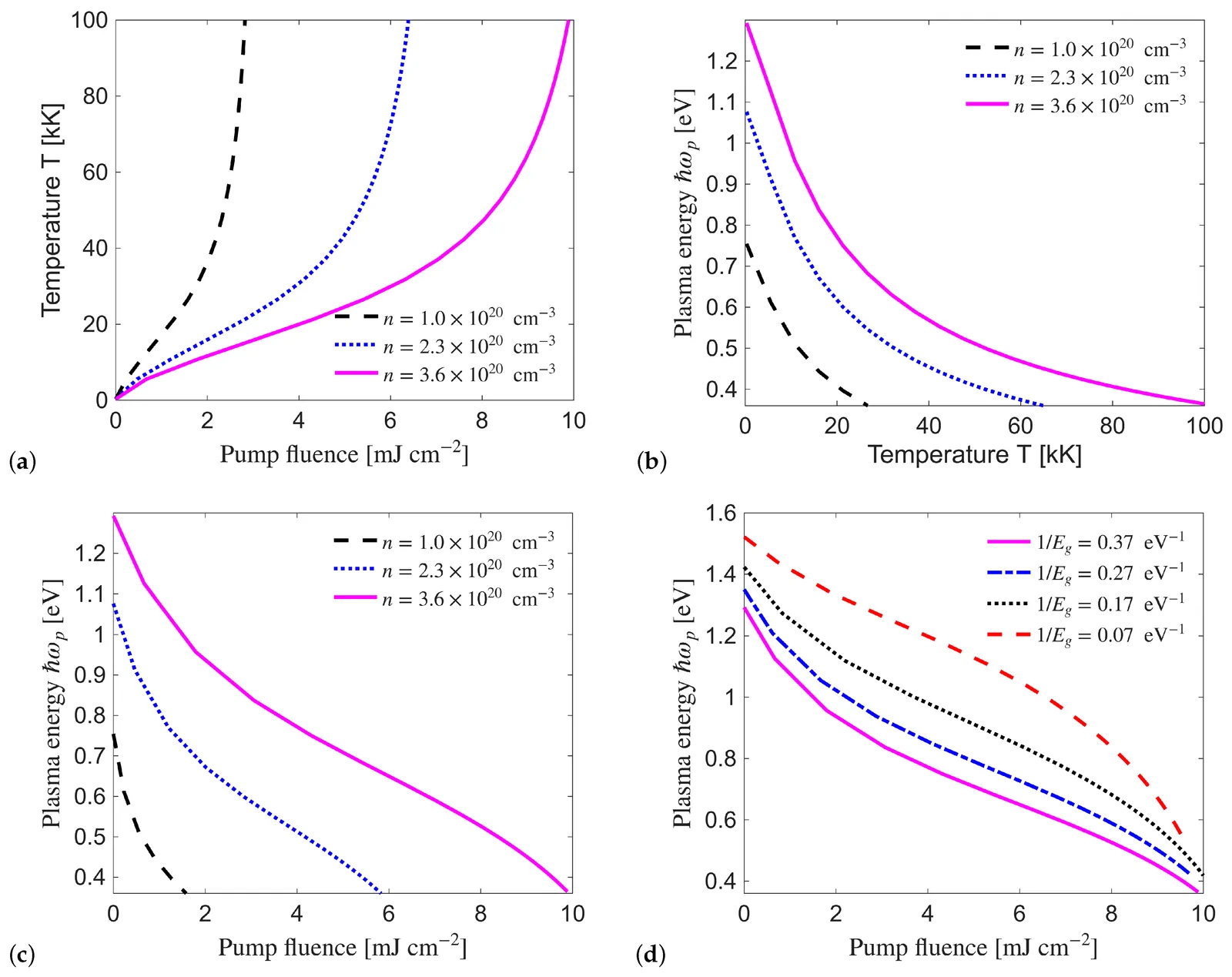
(**a**) Electron temperature *T* vs. pump fluence. (**b**) Plasma energy $\hbar \omega_{p}$ vs. electron temperature *T*. (**c**) Plasma energy $\hbar \omega_{p}$ vs. pump fluence. In panels (**a**–**c**), the LBSO carrier concentration *n* varies, and $1/E_{g}=0.37$ eV^−1^. (**d**) Plasma energy $\hbar \omega_{p}$ vs. pump fluence calculated for different conduction band nonparabolicities $1/E_{g}$ for $n=3.6\times 10^{20}$ cm^−3^.

Under near-infrared optical excitation, LBSO exhibits a reduction in plasma frequency arising from the nonparabolic nature of its conduction band introduced through the parameter $E_{g}$. As the carrier energy increases, the effective electron mass grows, modifying the carrier dynamics while the carrier concentration remains essentially unchanged. This effect gives rise to an intrinsic nonlinear response, enabling a plasma-frequency reduction of up to a factor of two. Using the values of $1/E_{g}=0.37\;{\mathrm{eV}}^{-1}$ and $m=0.19\;m_{0}$ for LBSO, we calculate μ(T), $\omega_{p}\left(T\right)$, and U(T) for the electron temperature *T* ranging from 300K to 100,000K and relate it to the pump fluence (Figure 3). Higher fluence is required to obtain the same electron temperature *T* for a higher LBSO carrier concentration *n*, which is explained by the need to deposit more energy if there are more carriers in the lattice (panel a). Plasma frequency $\omega_{p}$ can drop by half with realistic fluence, and the change is stronger for the lower carrier concentration *n* (panel b) and can be achieved with a lower pump fluence (panel c). Nonparabolicity $E_{g}$ enables the nonlinear response. Under variation in $E_{g}$, while plasma frequency $\omega_{p}$ experiences a slight increase when $1/E_{g}$ decreases, higher $1/E_{g}$ results in faster changes in plasma frequency $\omega_{p}$ (panel d).

LBSO is particularly attractive for high-speed optical modulation because of its ultrafast carrier dynamics and strong nonparabolicity-induced ENZ nonlinearity. The resulting induced perturbation of the carrier distribution enables sub-picosecond refractive-index modulation. Furthermore, the modulation remains effective over a broad spectral range due to intraband carrier transitions governed by femtosecond-scale scattering processes and the inherently broadband nature of Drude dispersion.

**Figure 4:**
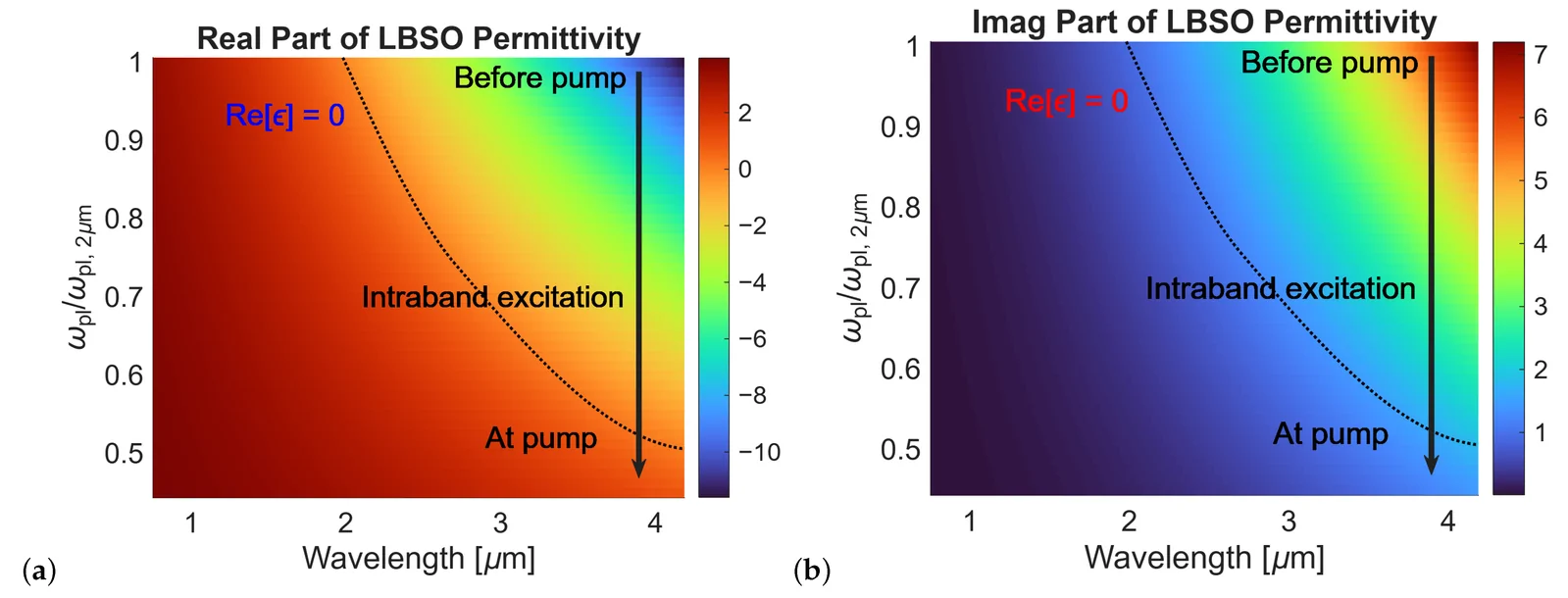
LBSO permittivity modulation under near-infrared optical pump: (**a**) Real part ℜ[ε] and (**b**) Imaginary part ℑ[ε]. An ultrafast optical pump reduces the plasma frequency, leading to an increase in ℜ[ε] and a decrease in ℑ[ε]. Black dashed lines on both panels indicate the epsilon-near-zero condition, that is, $ℜ[\varepsilon \left(\lambda_{\mathrm{ENZ}}\right)]=0$.

The optical response of LBSO is dispersive, and next we consider the material parameters that correspond to the crossover wavelength of 2.0μm. Optical pumping induces a substantial change in complex permittivity (Figure 4). In the unpumped state, LBSO exhibits a relatively lossy response: ℜ[ε]=3.00 and ℑ[ε]=0.11 at a wavelength of 1.00 μm and ℜ[ε]=−10.5 and ℑ[ε]=6.4 at a wavelength of 4.00 μm. Upon optical pumping, where the plasma frequency is reduced to half of its initial value, the permittivity changes to ℜ[ε]=3.78 and ℑ[ε]=0.03 at a wavelength of 1.00 μm and ℜ[ε]=0.60 and ℑ[ε]=1.5 at a wavelength of 4.00 μm, showing a significant reduction in optical losses. The state with $\omega_{\mathrm{pl}}=\omega_{\mathrm{pl},\;2.0\mu m}$, corresponding to the higher-loss condition, can be considered as the modulator’s off-state. The optically pumped state with $\omega_{\mathrm{pl}}=0.5\;\omega_{\mathrm{pl},\;2.0\mu m}$, which exhibits substantially lower losses, can be used in modulators as the on-state.

## 5. Metasurface Switching

**Figure 5:**
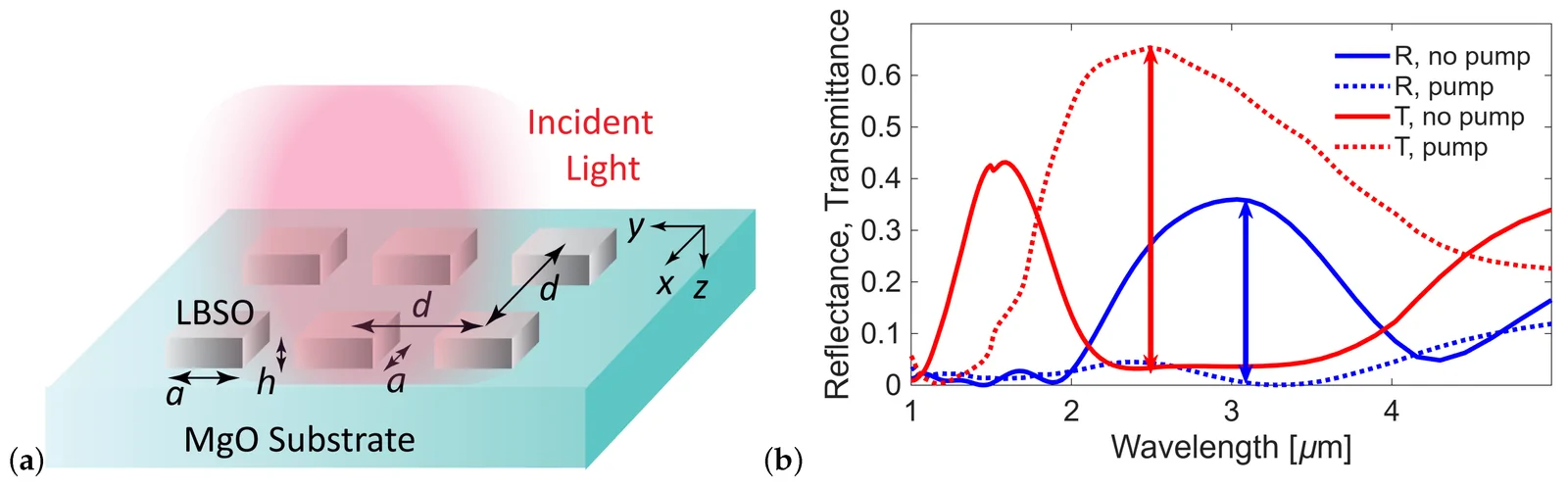
Switching of the metasurface response under a near-infrared optical pump. (**a**) Schematic of the LBSO nanoantenna arranged in the array on top of the MgO substrate. The nanoantenna dimensions are a×a×h, and the array period is *d* in both directions. (**b**) Change in reflectance and transmittance of the LBSO metasurface. Here, a=h=1μm, d=1.5μm, LBSO has a crossover wavelength of 2.0μm (that is $ℜ[\varepsilon \left(\lambda_{\mathrm{ENZ}}=2.0\;\mu m\right)]=0$), and the plasma frequency decreases by half under the pump. The double-headed arrows indicate the changes in the quantities at the wavelengths where these changes are most pronounced.

Using the properties of LBSO, let us analyze the optical switching behavior of the LBSO metasurface induced by near-infrared pumping, which modifies the free-carrier response and shifts the spectral position of the resonant features (Figure 5). The pronounced changes in reflectance and transmittance near the ENZ wavelength ($\lambda_{\mathrm{ENZ}}=2.0\;\mu m$) indicate the strong sensitivity of the metasurface modes to variations in plasma frequency. The largest change in reflectance is 0.35 at the wavelength of 3.1 μm, and the largest change in transmittance is 0.62 at the wavelength 2.5 μm. Because the optical response of LBSO is governed by the interplay between localized nanoantenna resonances and ENZ dispersion, a reduction in plasma frequency alters the balance between scattering and transmission channels. This behavior demonstrates the potential of ENZ-based LBSO metasurfaces for ultrafast and reversible modulation of mid-infrared light through carrier-density control.

The absolute transmittance and the quality factor of the metasurface resonances can be improved through optimization of the nanoantenna geometry and LBSO material parameters. In particular, reducing material absorption and carrier scattering can narrow the resonance linewidth and increase its quality factor, while appropriate control of the radiative coupling can maintain efficient transmission. At the same time, the resonance should retain sufficient overlap with the ENZ region to preserve its sensitivity to pump-induced changes in the LBSO permittivity. Thus, optimization of the balance between radiative loss, material absorption, and ENZ-induced field enhancement provides a pathway toward simultaneously achieving high absolute transmission and strong modulation depth.

The ultimate speed of the optical modulation is governed not by the instantaneous electromagnetic response itself, but by the relaxation of the photoexcited carrier distribution. Following optical excitation, carrier–carrier interactions establish a nonequilibrium distribution on ultrafast timescales, while subsequent carrier–phonon interactions and energy relaxation govern the recovery toward equilibrium. Thus, carrier thermalization and, in particular, carrier cooling provide the fundamental physical limits on the recovery time and repetition rate of all-optical modulation. The present collisionless model describes the field-induced modification of the carrier distribution but does not explicitly include these scattering and relaxation processes.

Compared with commonly used TCOs such as ITO and AZO, LBSO offers several potentially attractive characteristics for tunable infrared photonics. In particular, LBSO can exhibit relatively high carrier mobility while maintaining a broad range of electrically and optically accessible carrier concentrations, enabling the ENZ wavelength to be tuned over a comparatively broad spectral range. LBSO also avoids the use of indium and has demonstrated good thermal stability [50], which may be beneficial for devices operating at elevated temperatures or optical powers. These properties make LBSO a promising complementary platform for infrared switching and modulation, although the practical device performance remains strongly dependent on film quality, carrier scattering, and device geometry.

A direct quantitative comparison of the modulation depth, insertion loss, and switching speed of LBSO with ITO and AZO requires nominally identical film thicknesses, metasurface geometries, excitation fluences, and probe conditions. Such a controlled comparison is beyond the scope of the present work. Nevertheless, LBSO provides the same ENZ-based mechanism for strong optical modulation while offering a broad range of ENZ wavelengths through control of the carrier concentration. In addition, the relatively high carrier mobility reported for LBSO (83–86 cm^2^/Vs, compared with about $23\;{\mathrm{cm}}^{2}/\mathrm{Vs}$ for ITO and $21\;{\mathrm{cm}}^{2}/\mathrm{Vs}$ for AZO [50]) is favorable for reducing free-carrier damping and optical losses.

## 6. Conclusions

This work examined the nonlinear optical response of LBSO using a collisionless Boltzmann framework, accompanied by a demonstration of LBSO films with variable properties upon pulsed laser deposition. We obtained high-quality LBSO thin films with controlled optoelectronic properties and a well-defined ENZ wavelength. The analytical model developed demonstrates that optical excitation modifies the electron distribution function, thereby changing both the effective plasma frequency and the dielectric permittivity. The results showed a possibility of a substantial reduction in the plasma frequency with increasing excitation strength, accompanied by noticeable changes in the material’s spectral response. These findings highlight the strong interplay between carrier dynamics and electromagnetic properties in LBSO and provide a theoretical foundation for exploiting its nonlinear behavior in tunable infrared and terahertz photonic devices. We show that near-infrared optical pumping dynamically reconfigures the LBSO metasurface, resulting in pronounced changes in its reflectance and transmittance spectra near the ENZ regime. More broadly, the presented approach can be extended to the study of nonlinear phenomena in other transparent conducting oxides and ENZ materials.

The planar nature of LBSO thin films also provides a pathway toward integration with established silicon photonic platforms. An LBSO active layer could be incorporated adjacent to a silicon waveguide, allowing the guided optical mode to interact with the ENZ region, while fiber coupling could be provided using conventional edge- or grating-coupling structures. Such integration would introduce additional design considerations, including the overlap between the guided mode and the LBSO layer, propagation and absorption losses, thermal management, and the precise alignment of the ENZ wavelength with the operating wavelength of the photonic circuit. In addition, the fabrication process must maintain the optical quality and carrier concentration of the LBSO layer while remaining compatible with the underlying silicon photonic structures. Optimization of these parameters represents an important direction for future integrated LBSO photonic devices.

## Figures and Tables

**Table 1:** Optical properties of LBSO films deposited at various conditions, as well as Drude parameters extracted from fitting. $\lambda_{\mathrm{ENZ}}$ corresponds to the wavelength where the real part of the permittivity crosses zero (ℜ[ε]=0), with ‘ENZ’ standing for ‘epsilon near zero’. In our notation, the plasma frequency is expressed as $\omega_{p}=2\times 10^{15}$ s^−1^, corresponding to the plasma energy $\hbar \omega_{p}=1.316$ eV.

#	$\varepsilon_{\infty }$	$\omega_{p}$ ($\times 10^{15}$ s^−1^)	γ ($\times 10^{14}$ s^−1^)	$m^{*}/m_{0}$	*n* ($\times 10^{20}$ cm^−3^)	$\lambda_{\mathrm{ENZ}}$ (nm)
1	4.05	1.96	2.07	0.30	3.62	1980
2	4.26	1.94	3.296	0.29	3.54	2140
3	4.30	1.81	3.730	0.29	3.07	2384
4	4.23	1.58	3.48	0.29	2.36	2750
5	4.00	1.20	1.96	0.29	1.32	3320
6	4.00	1.24	4.33	0.28	1.46	4000
7	4.00	1.14	8.00	0.28	1.15	4790
8	4.00	1.09	12.5	0.27	1.01	5600

## Data Availability

The data that support the findings of this study are available from the corresponding author upon reasonable request.
